# Prevalence of impacted and supernumerary teeth in the North Indian population

**DOI:** 10.4317/jced.51284

**Published:** 2014-04-01

**Authors:** Santosh Patil, Sneha Maheshwari

**Affiliations:** 1 Dept of Oral Medicine and Radiology, Jodhpur Dental College, Jodhpur National University, Jodhpur (Raj), India

## Abstract

Objectives: Dental impaction is a very frequent problem. Supernumerary teeth, or hyperdontia, are the presence of additional teeth to the normal series in the either of the dentition. The presence of impacted and/or supernumerary teeth can cause various complications. The objective of the present study was to determine the prevalence of impacted and supernumerary teeth in the North Indian population. Study Design: The panoramic radiographic records of 4750 patients attending the Department of Oral Medicine and Radiology, Jodhpur Dental College General Hospital between September 2008 to December 2012 were examined for this retrospective study. The ages of the patients ranged from 8 to 72 years, with a mean of 34.6 years. Results: A total of 798 (16.8%) patients presented with 1126 impacted teeth. Impacted canines were the most prevalent (9.7%), followed by impacted premolars (4.3%). Supernumerary teeth (1.6%) and impacted molars (1.2%) were less prevalent. Among the 842 impacted teeth, the most frequently affected teeth were the canines (56.7%), followed by premolars (27.8%), and supernumerary teeth (9.3%), while the prevalence of impacted molars was quite lower (6.2%). Conclusion: The most frequently impacted teeth were maxillary canines and the mesiodens were the most common supernumerary tooth. The early diagnosis of supernumerary and impacted teeth is essential to prevent malocclusion and malalignment of permanent teeth demonstrating the importance of panoramic radiographs in their detection.

** Key words:**Impacted, supernumerary, prevalence, canines, mesiodens.

## Introduction

Impacted teeth are those that remain unerupted and retained or that are partially erupted based on clinical and radiographic evaluation (1). Failure of the eruption of permanent teeth is commonly seen dental anomaly (2). These impacted teeth pose many problems like affecting tooth movement, esthetics, and functional consequences. The eruption of permanent teeth is a complex event, which is genetically based. The eruptive movement of the tooth germ takes place at a predetermined time and route, thus enabling the tooth to find its antagonist at a predetermined occlusal level. The successful development of permanent teeth also is in sync with the forward and lateral growth of both the jaw bones, which compensates for the difference in size of the dentition in both bones. As the eruption process is so complex, it leads to various complications including tooth retardation or failure of eruption (2).

A supernumerary tooth is a dental anomaly of number characterized by the presence of an additional tooth in the normal series. Supernumerary tooth is more common in the permanent dentition than in the primary dentition (3). The etiology is not known. Various theories have been suggested for the presence of supernumerary tooth, such as dichotomy of the tooth bud, hyperactivity of the dental lamina and a combined effect of genetic and environmental factors (4,5). Supernumerary teeth may also occur in association with syndromes like cleft lip and palate, Cleidocranial dysplasia, Down’s syndromes, etc (6). While supernumerary tooth may be found in any region of the dental arch, the most common site is the midline between the 2 maxillary central incisors, where it is referred as mesiodens. They account for 80% of all supernumerary teeth (3). Asymptomatic unerupted mesiodens may be discovered during clinical and radiological examination of the maxillary anterior region by periapical and panoramic radiographs.

Radiographic and clinical examination may reveal impacted or supernumerary teeth. Extraction of these teeth depends on clinical and radiographic diagnosis showing that the tooth would erupt or not, there would be lack of space in the dental arch to accommodate it and also when pathological and neoplastic processes are associated with it (2). The prevalence of impacted and supernumerary teeth in different populations and ethnic groups has been the subject of several studies. The prevalence of impacted teeth, excluding third molars, has been reported to vary between 5.6 to 18.8%. The prevalence of supernumerary teeth varies between 0.3 and 3.8% (1,2,7). The objective of the present study was to determine the prevalence of impacted and supernumerary teeth in the North Indian population.

## Material and Methods

The panoramic radiographs of 4750 patients attending the Department of Oral Medicine and Radiology, Jodhpur Dental College General Hospital between September 2008 to December 2012 were examined for this retrospective study. Patients’ dental records and radiographs were examined in order to detect the impacted canines, impacted premolars, impacted molars (except third molars), and supernumerary impacted teeth. The ages of the patients ranged from 8 to 72 years, with a mean of 34.6 years. All panoramic radiographs were taken with the Dentsply Gendex Orthoralix 9200 (Dentsply Asia, Milford, US), and the magnification factor was 1.23. One group of researchers examined the radiographs at the same time on standard light boxes to determine the impacted tooth.

A tooth was defined as impacted when the tooth was obstructed on its path of eruption by an adjacent tooth, bone, or soft tissue. Taking into account the mean eruption time, the teeth were considered as impacted when they remained in the jaw for a minimum of 2 years after the corresponding mean age of eruption (8). A supernumerary tooth is an additional tooth in the normal series, erupted or unerupted, and may resemble or is unlike the other teeth of the group to which it belongs (2). After the examination of the patient records, patients who exhibited any pathological conditions, trauma or fracture of the jaw that might have affected the normal growth of permanent dentition or any hereditary diseases or syndromes were excluded from the study. The observations were entered and analyzed using the computer program, SPSS 12 (SPSS Inc. Chicago, USA). The differences between the groups were tested using the Chi-square test.

## Results

There were 2465 males (51.9%) and 2285 females (48.1%), with an age range of 8-72 years and a mean age of 34.6 years ( Table 1). Out of the 4750 patients that were examined, a total of 798 (16.8%) patients presented with 1126 impacted teeth, of which 425 (53.2%) were males and 373 (46.8%) females. The prevalence of impacted teeth was not statistically significant among the sexes (*p*>0.05). 511 of the patients had at least 1 impacted tooth, 249 had 2 impacted teeth and only 38 patients had >3 teeth that were impacted ( Table 2). The number of impacted teeth was not statistically significant in relation to gender (*p*>0.05). Impacted canines were the most prevalent (9.7%), followed by impacted premolars (4.3%). Supernumerary teeth (1.6%) and impacted molars (1.2%) were less prevalent ( Table 3). Impacted canines were commonly seen in females while supernumerary and impacted premolars and molars were more common in males. This gender specific increased prevalence of impacted teeth was statistically significant (*p*<0.05). No impacted incisors were noted in any of the patients.

**Table 1:**
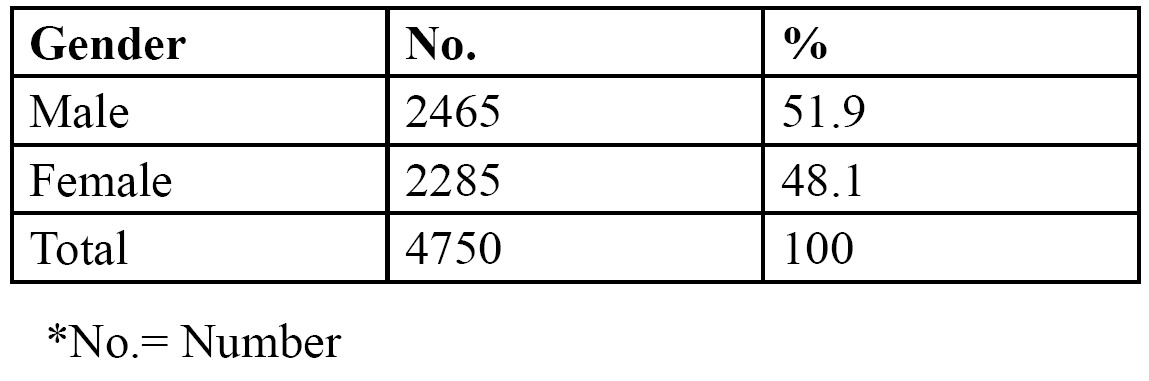
Distribution of patients according to gender.

**Table 2:**
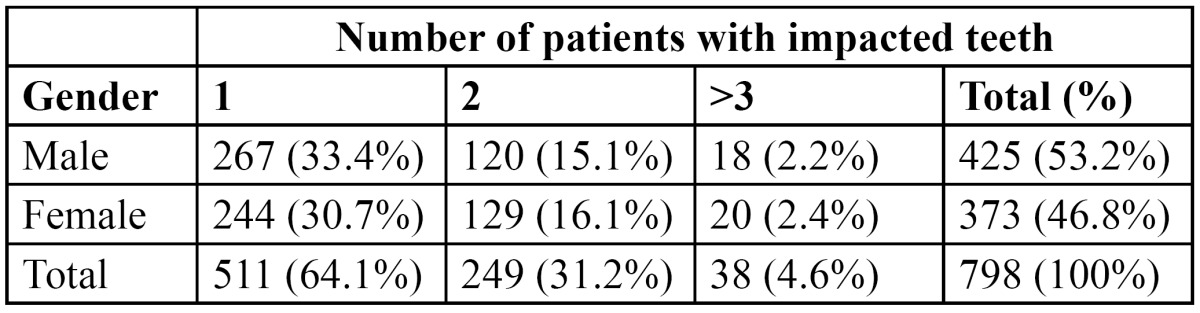
Distribution of patients with impacted teeth according to gender and number of impacted teeth.

**Table 3:**
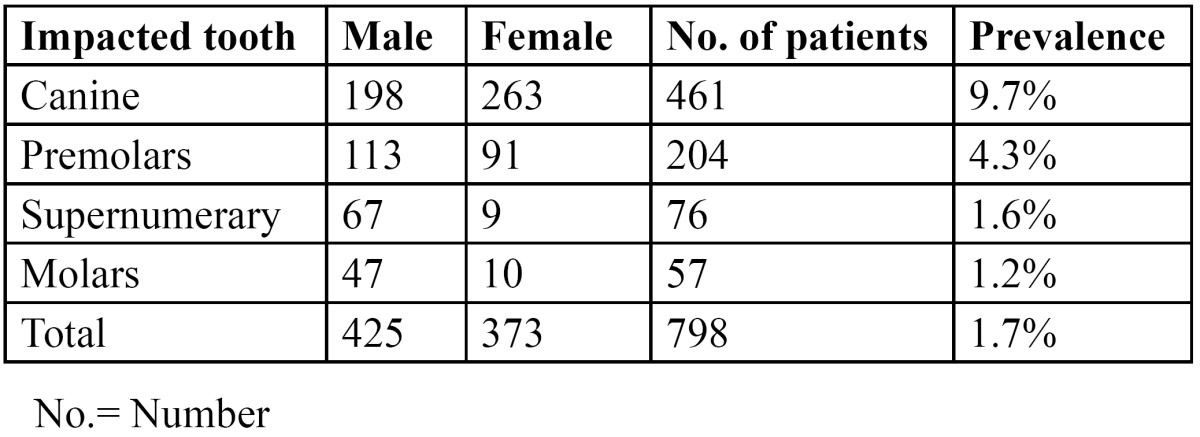
Distribution of patients according to the type of impacted teeth and prevalence of impacted teeth.

Among the 1126 impacted teeth, the most frequently affected teeth were the canines (56.7%), followed by premolars (27.8%), and supernumerary teeth (9.3%), while the incidence of impacted molars was quite lower (6.2%). Mostly the maxillary canines (87.5%) were impacted, with only 66 impacted canines in the mandibular arch. There was no statistically significant difference between the type of impacted tooth and gender (*p*<0.05). While most of the impacted molars and premolars were located in the mandible ( Table 4). However, this was not statistically significant (*p*<0.05). Most of the patients with impacted teeth had only 1 tooth that was impacted. A total of 76 patients had supernumerary tooth, most commonly in between the 2 maxillary central incisors. 56 patients had mesiodens, 8 patients had supernumerary tooth in the lateral incisor region and 12 patients had supernumerary tooth in the premolar and molar region. The patients with supernumerary tooth were younger than the patients with impacted tooth.

**Table 4:**
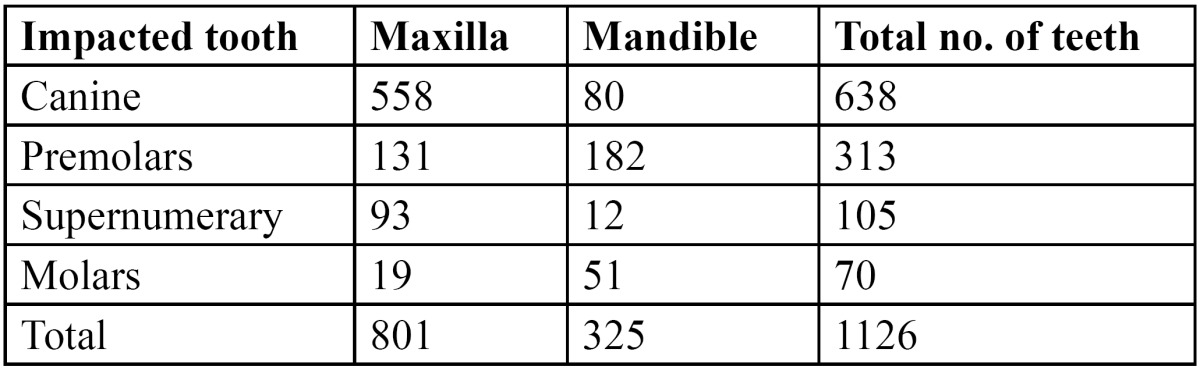
Distribution of impacted teeth according to their location.

## Discussion

Panoramic radiographs are largely used as an important diagnostic tool in dental practice. Dental and bony injuries, presence of cysts and tumors, and dental anomalies of number, size and shape, are examples of alterations that can be conveniently observed on a panoramic radiograph. The use of panoramic radiographs to identify developmental disturbances in children around the ages of 6 to 9 years has been indicated (9). To ensure the diagnostic validity of the study all patients and their radiographic findings were studied thoroughly. The present results indicate that the prevalence of tooth impaction in North Indian population is within the range of 5.6-18.8% as reported in other studies (2). Our data shows the prevalence of tooth impaction to be 16.8%.

Dental impaction is frequently found in dental practice in teenagers and adults with the third molar and in children with the upper canines (8). The present study has shown the prevalence of maxillary canine impaction to be 9.7%, which is much higher than the range of 0.2% to 2.5% reported in other studies (10). The canine tooth has a complicated eruption pattern and is one of the last teeth to erupt in dental arch. According to these conditions, this tooth may not have an eruption process in a natural way (11). We found that canines were the most commonly impacted teeth, which is in line with the studies of Fardi *et al.* (2), who reported a prevalence of 8.8% in the Greek population. In a similar study, 4898 Saudi patients aged 13 years and older were examined, who showed a prevalence of 3.6% with at least one impacted cuspid (12). Another study that analyzed 1858 patients of the 11-18 year age group presented for orthodontic treatment, revealed 101 cases of impacted canines with a prevalence of 5.43% (13). Aydin *et al.* (14) reported an incidence of 3.58%, which was lower than the findings of the present study. These results indicate that the incidence of canine impaction may be higher in some populations. The different results may be attributed to the racial differences and differences in the methodology of the study. The Japanese have shown to have the lowest frequency as reported in the literature, where the anomaly occurred in only 0.27% of the study population. Similar to these findings, study of a large series of full mouth dental radiographs in the USA revealed a figure of 0.92% (7). While Brin *et al.* (15) in their study of an Israeli population, found a level of 1.5%.

Maxillary impactions are believed to occur 10-20 times more frequently than mandibular (16). Mandibular canine impaction occurs in very low incidence and there are limited numbers of studies revealing its frequency of occurrence. Impacted canines of the mandible are very rare in occurrence. In the study by Shah *et al.* (17), 8 unerupted mandibular canines were found in 7886 individuals, and in another study 11 impacted mandibular canines were found in 5000 individuals, resulting in an incidence of 0.10% (18). Although there was no difference in the sex distribution for impacted canines in our study, the male to female prevalence rate ratios was 1:1.3. This result is within the range from 1:1.3 to 1:3.2 as reported in other similar studies, indicating a higher prevalence of impacted canines among females (19). There was no statistically significant difference between the impacted tooth type and gender (*p*<0.05), similar to the study of Fardi *et al.* (2).

Very few studies have been done regarding impacted premolars. It has been concluded from the results of these studies that premolar impaction is rare, with the prevalence ranging from 2.1-2.7% (8,20,21). The results of the present study are however higher, with a prevalence of 4.3%. The present study indicated that the mandibular premolars were the frequently impacted tooth. While it was shown in a study that maxillary premolars remained impacted more frequently (22). The impacted molars is a very rare abnormality, which is in conjunction with the low incidence (1.2%) found in the present study (23). Similar with the findings of other studies, we observed increased prevalence in male patients, which as suggested may be due to a genetic component (2,23). However, this increased prevalence in males was not statistically significant (*p*<0.05). Impacted teeth may lead to various complications such as abscess of the tooth or gums, malocclusion of teeth, recurrent infection of the impacted tooth, chronic discomfort in the mouth and increased plaque and debris accumulation and food entrapment between the tooth and the soft tissues leading to pericoronitis (22).

Supernumerary teeth are not an uncommon finding in dental practice. Supernumerary teeth or hyperdontia describes an excess in tooth number. The prevalence of hyperdontia is reported to lie between 1-3% in permanent dentition and is considerably rarer in the primary dentition. The aetiology of supernumerary teeth is unknown, and several theories have been suggested. Clinical complications are not uncommon in patients with supernumerary teeth. Tooth displacement and failure of eruption are the most frequently seen complications (24). The reported prevalence of supernumerary impacted teeth (1.6%) in the present study falls within the range of 0.1-3.8% as reported earlier (2,26). Bäckman and Wahlin (25) found 14 cases with one supernumerary tooth in a study in the Caucasian population. They also noted that the majority of the supernumerary teeth were mesiodens, which is in line with the present study. Another study of 2,393 Saudi Arabian children found the prevalence of supernumerary tooth to be 0.5% (26). In contrast to the 2:1 ratio between males and females reported in Caucasians (27), in the present study the sex ratio was much higher, being 7.2:1 in favor of males. Most supernumerary teeth are impacted and asymptomatic and diagnosed incidentally during radiographic examinations. Panoramic radiograph is thus essential for the early detection of supernumerary teeth.

To determine the actual prevalence of tooth impaction, a representative and randomized sample of the general population is required. Here, radiographic examination from specific populations seems to be the most common practical approach, which will inevitably involve the risk of bias in the data analysis.

The present study showed that the prevalence of tooth impaction was 16.8%. This indicates that tooth impaction is a common dental anomaly. Impacted canines were the most commonly impacted teeth (9.7%) and molars were found to be least impacted (1.2%). The dissimilarities may be attributed to the sample selection, method of the study and area of patient selection, which suggest racial and genetic differences. Early detection of dental development anomalies is very important, as they may lead to many unseen complications.
